# End TB strategy: the need to reduce risk inequalities

**DOI:** 10.1186/s12879-016-1464-8

**Published:** 2016-03-22

**Authors:** M. Gabriela M. Gomes, Maurício L. Barreto, Philippe Glaziou, Graham F. Medley, Laura C. Rodrigues, Jacco Wallinga, S. Bertel Squire

**Affiliations:** Liverpool School of Tropical Medicine, Liverpool, UK; CIBIO-InBIO, Centro de Investigação em Biodiversidade e Recursos Genéticos, Universidade do Porto, Vila do Conde, Portugal; Instituto de Matemática e Estatística, Universidade de São Paulo, São Paulo, Brazil; Centro de Pesquisas Gonçalo Moniz, FIOCRUZ, Salvador, Brazil; Instituto de Saúde Coletiva, Universidade Federal da Bahia, Salvador, Brazil; World Health Organization, 1211 Geneva 27, Switzerland; London School of Hygiene and Tropical Medicine, London, UK; Department of Infectious Diseases Epidemiology, National Institute of Public Health and the Environment, PO Box 1, 3720 BA Bilthoven, The Netherlands; Department of Medical Statistics and Bioinformatics, Leiden University Medical Center, PO Box 9600, 2300 RC Leiden, The Netherlands

**Keywords:** Tuberculosis, Heterogeneity, Cohort selection, Social inequality, Intervention impact

## Abstract

**Background:**

Diseases occur in populations whose individuals differ in essential characteristics, such as exposure to the causative agent, susceptibility given exposure, and infectiousness upon infection in the case of infectious diseases.

**Discussion:**

Concepts developed in demography more than 30 years ago assert that variability between individuals affects substantially the estimation of overall population risk from disease incidence data. Methods that ignore individual heterogeneity tend to underestimate overall risk and lead to overoptimistic expectations for control. Concerned that this phenomenon is frequently overlooked in epidemiology, here we feature its significance for interpreting global data on human tuberculosis and predicting the impact of control measures.

**Summary:**

We show that population-wide interventions have the greatest impact in populations where all individuals face an equal risk. Lowering variability in risk has great potential to increase the impact of interventions. Reducing inequality, therefore, empowers health interventions, which in turn improves health, further reducing inequality, in a virtuous circle.

**Electronic supplementary material:**

The online version of this article (doi:10.1186/s12879-016-1464-8) contains supplementary material, which is available to authorized users.

## Background

The World Health Organization (WHO) has defined a new strategy for tuberculosis (TB) prevention, care and control – the End TB Strategy – and set, among its targets, to reduce the incidence rate of the disease by 90 % in 20 years (2015–2035) [1]. There is some urgency for TB control as drug resistance increases and populations become more mobile – TB becomes more difficult to treat while effectively infecting the world’s poorest communities no matter where they live. Consequently, WHO estimates that the reduction in global incidence must accelerate from the current 2 % per year [2] to meet new targets, and advocates strong social protection in addition to universal health care [3]. The marked heterogeneity in TB risk makes its dynamics particularly responsive to synergistic policies, such as targeted social measures or targeted HIV treatment to reduce variation, combined with TB interventions targeting the whole population to reduce disease. Adding to previous studies, which have assessed targeting control at the poor [4] or implementing population-wide measures with fixed stratification of disease risk [5], here we examine how the impact of universal coverage interventions, such as a hypothetical vaccine against TB, depends on the underlying risk distribution in a population. Universal coverage interventions have greater impact on disease in populations where all individuals face similar risks, indicating that reducing inequality empowers existing disease control tools.

### Misuse of population averages

**Fig 1:**
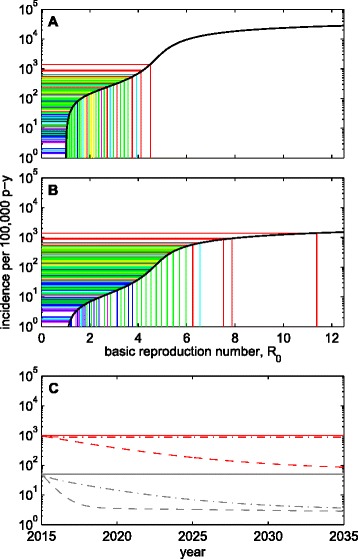
Global tuberculosis incidence per 100,000 person-years. **a**, **b**, Endemic equilibrium states for model in appendix (*black curves*: **a**, homogeneous; **b**, heterogeneous). Colored lines represent the TB incidences reported by WHO in all countries, and associated *R*_0_. Countries are color-coded by WHO region: African (*red*); South-East Asia (*yellow*); Eastern Mediterranean (*cyan*); Western Pacific (*green*); Europe (*blue*); The Americas (*magenta*). **c**, Simulation of a vaccine that halves susceptibility to infection and reduces reactivation rate by 90 %. Dashed and dash-dotted curves correspond to populations with variance-to-mean ratios of 0 (homogeneous) and 20 (heterogeneous), respectively, in two epidemiological settings: baseline incidence of 1000 per 100,000 person-years (*red*); and 50 per 100,000 person-years (*grey*)

In tuberculosis, it is well known that some groups have much higher risk to acquire infection and develop disease than the majority of the population: incarcerated prisoners (20 times higher in Brazil [6]), persons living with HIV (8 times higher in a South African community [7]), geographical hotspots within urban settings (3 times higher in people living in the poorest areas in Rio de Janeiro [8]), among others. Behind these differences are differences in basic parameters, which measure how frequently each person is likely to meet an infectious case of TB and become infected, as determined by the way social mixing patterns combine with individual vulnerabilities [4]. These effective contact rates are then averaged and scaled into the so-called basic reproduction number, *R*_0_, defined as the average number of secondary disease cases caused by an average infectious person in a totally susceptible population. Similarly, the incidence of TB in the population is usually presented as a single number, which implicitly represents a weighted average of the incidences in groups of different sizes and relative risks. All this averaging would be fine if disease probabilities were linearly related with exposure intensity, but incidence describes a non-linear relationship with *R*_0_ (Fig. 1). This relation has a concave form due to the stronger depletion of susceptible hosts at higher transmission intensities. As a result, averaging incidences leads to underestimated *R*_0_ and overoptimistic control expectations at the population level as recently demonstrated by a theoretical study applicable to infectious diseases more generally [9].

### Why there is a tendency to underestimate control efforts

To illustrate the idea, we use a transmission model for TB, developed previously [10] and replicated in the appendix. In Fig. 1a, b incidence data for different countries are shown in a colour scheme defined by WHO region [2], superimposed on how rates of TB incidence relate to *R*_0_ according to two realisations of the model: a homogenous assumption, where all individuals experience the same rates of infection, disease progression and transmission (Fig. 1a); and a heterogeneous implementation where 4 % of individuals have a higher risk to contract the infection and infect others [8, 10, 11] (Fig. 1b). All parameters satisfy the same mean values across the two model realisations, allowing comparison and demonstrating how heterogeneity in risk has a striking ability to modify the correspondence between incidence and *R*_0_. More specifically, to a given incidence, the heterogeneous model associates a reproduction number that is higher than that given by the corresponding homogeneous counterpart, resulting in greater resilience to universal coverage control efforts.

For intuition, consider a population with some distribution of TB risk. The first people to be infected are those with higher risk, leaving behind a susceptibility pool with an average risk that decreases over time, by a process of cohort selection [12, 13], until the system reaches equilibrium. Thus cohort selection acts effectively to suppress disease incidence, the more so the higher the transmission intensity. Now suppose that TB transmission is formulated into a model that collapses the risk distribution into its mean value. Cohort selection no longer occurs because all individuals in this model have the same risk, and therefore the system sustains a higher disease incidence by the time it reaches equilibrium. It follows that when two populations present the same overall disease incidence, we should expect a higher average risk where inequality is higher, but this is not revealed in overall disease incidence. Interestingly, any universal coverage intervention that reduces (directly) transmission potential also reduces (indirectly) the suppression imposed by cohort selection, and this effect is greater where inequality is higher. Therefore, intervention impact is effectively lower under risk inequality and this is aggravated as the intervention progresses.

### Universal coverage intervention impact

In Fig. 1c, we introduce a universal coverage intervention – for example a vaccine against TB. We consider settings that, in 2015, present mean incidences of 1000 per 100,000 person-years (red), as in the highest burden African countries, and a more common scenario of 50 per 100,000 person-years (grey). For each setting, we contrast the time course of incidence rates under a vaccine that effectively halves everybody’s susceptibility to infection and reduces reactivation rate by 90 %, assuming that the risk distribution is homogeneous (dashed) or heterogeneous (dash-dotted). The impact of the intervention is consistently higher under homogeneous risks, although the magnitude of the difference can vary between settings. In this case, the higher incidence setting shows greater difference in predicted impact: over 20 years, the vaccine reduces incidence by 91 % or just by 10 %, depending on whether the variance-to-mean ratio in risk is 0 (homogeneous) or 20 (heterogeneous) (Fig. 1c, red). The same simulation for a population presenting a lower baseline incidence of 50 per 100,000 person-years leads to more consistent reductions by, respectively, 94 % or 92 % after 20 years although the reduction is slower when heterogeneity is accounted for (Fig. 1c, grey).

**Fig 2:**
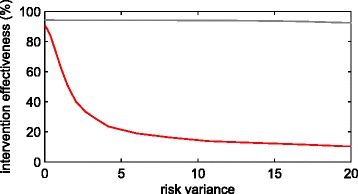
Impact of simulated vaccine decaying with the variance-to-mean ratio of disease risk. In all cases the vaccine is given to all individuals to halve susceptibility to infection and reduce reactivation rate by 90 %. Two epidemiological settings are considered: baseline incidence of 1000 per 100,000 person-years (*red*); and 50 per 100,000 person-years (*grey*)

Figure 2 summarises how the 20-year impact of the simulated vaccination programme decays with risk heterogeneity, considering a range of variance-to-mean ratios between 0 and 20. An extended impact analysis over baseline incidences is provided in the appendix. Additional file 1: Figure S1 shows expected incidences 20 years since the intervention has started, as well as a projection of the same measures when new equilibria are eventually established. Time to approach equilibrium varies considerably across settings, appearing particularly high in those settings which are closer to elimination, thus indicating the importance of accurate time descriptions as elimination is approached.

## Conclusions

The analysis provided here has profound implications for the assessment of progression towards control targets, such as those recently set by the WHO [1]. We have used a hypothetical vaccine to quantify how heterogeneity in individual disease risk can compromise the performance of control programmes. In this exercise, the recommended target of 90 % reduction in incidence by 2035 can be comfortably met in settings where the incidence is 50 per 100,000. In contrast, when incidence is much higher, e.g. 1000 per 100,000, then considerably more effort is required. Averaging model parameters over groups of very different risk tends to unrealistically assign low values to transmission indices, such as *R*_0_, leading to overoptimistic control expectations especially in high incidence regions.

In highlighting the sensitivity of programme outcomes to the actual distribution of disease risks in populations, we hope to stimulate a concerted action by the research community to optimise analytic tools. Incidence data at finer stratification are needed for the construction of strategic models that better capture heterogeneities in transmission. Models and targets need to take explicit account of risk inequalities and their determinants. Meanwhile, as shown here, policies that reduce risk inequalities, whether social [14, 15] or biomedical [16], are expected to boost the impact of population-wide interventions that target tuberculosis. Any disease specific control programme that ensures health interventions to work better for those at greater risk [17], contributes to the huge task of making populations more homogeneous, besides the more immediate returns in terms of reducing the target disease. Social protection and poverty alleviation actions [18, 19] can then synergise with these programmes to increase the power of already existing disease control tools.

## Additional file

Additional file 1: Mathematical model and extended intervention impact analysis. (PDF 422 kb)

## Abbreviations

WHO: World Health Organization
TB: tuberculosis
