# Supplementary material for: End TB strategy: the need to reduce risk inequalities
Source: BMC Infect Dis. 2016 Mar 22;16:132. doi: 10.1186/s12879-016-1464-8 (PMC4802713; doi:10.1186/s12879-016-1464-8)
Supplement: Additional file 1: — Mathematical model and extended intervention impact analysis. (PDF 422 kb) [file 12879_2016_1464_MOESM1_ESM.pdf]

Supplementary Appendix - End TB strategy: the need to reduce risk inequalities

Mathematical model

We consider a previously published[1] tuberculosis transmission model:

$$\frac{dS_i}{dt} = p_i\mu - \lambda_i S_i - \mu S_i$$

$$\frac{dP_i}{dt} = \lambda_i S_i + \sigma\lambda_i L_i - (\delta + \mu) P_i$$

$$\frac{dI_i}{dt} = \phi\delta P_i + \omega L_i - (\tau + \mu) I_i$$

$$\frac{dL_i}{dt} = (1-\phi)\delta P_i + \tau I_i - \sigma\lambda_i L_i - (\omega + \mu) L_i,$$

where subscripts 1,2, denote low and high connectivity (risk) groups, and within each group individuals are classified - according to their infection history - into susceptible (S_i), primary infection (P_i), latent (L_i), and active pulmonary tuberculosis (I_i). The model parameters along with their typical values used herein are listed in Table S1. The force of infection upon naive individuals is $\lambda_i = (\kappa_i / \langle\kappa\rangle)\beta(\kappa_1 I_1 + \kappa_2 I_2)$, the basic reproduction number is:

$$R_0 = \left[\frac{\omega + \mu}{\mu(\tau + \omega + \mu)}\right]\left[\frac{\phi\delta}{\delta + \mu} + \frac{(1-\phi)\delta\omega}{(\delta + \mu)(\omega + \mu)}\right]\frac{\langle\kappa^2\rangle}{\langle\kappa\rangle}\beta,$$

and the effective reproduction number in a population where everyone has been exposed and developed partial immunity is:

$$R_e = \left[\frac{\phi\delta}{(\delta + \mu)(\tau + \mu)}\right]\frac{\langle\kappa^2\rangle}{\langle\kappa\rangle}\sigma\beta$$

The model admits an endemic equilibrium when $R_0 > 1$, which has been calculated[1] for both the two risk-group version and its mean field approximation, and plotted in Figure 1A,B.

Table S1. Parameters for tuberculosis transmission model.

Symbol	Definition	Value
β	Transmission coefficient	varying (yrs^{-1})
μ	Death and birth rate	1/70 yrs^{-1}
δ	Rate out of primary TB state	12 yrs^{-1}
ϕ	Fraction progressing from primary to active TB	0.05
σ	Reinfection factor	0.5
ω	Rate of relapse	0.001 yrs^{-1}
τ	Rate of successful treatment	2 yrs^{-1}
k_i	Low and high individual risk relative to population average	0.0875; 22.9
p_i	Proportion of individuals in low and high risk groups	0.96; 0.04

Simulating an intervention

The same model is then used as a basis to simulate an intervention that reduces the force of infection upon naive individuals to $\sigma\lambda_i$ (this is, conferring them with a degree of partial immunity as if they had been previously exposed to the mycobacterium) and the reactivation rate to $\omega/10$. A population under such intervention is then modeled as:

$$\frac{dS_{vi}}{dt} = p_i \mu - \sigma\lambda_{vi} S_{vi} - \mu S_{vi}$$
$$\frac{dP_{vi}}{dt} = \sigma\lambda_{vi} S_{vi} + \sigma\lambda_{vi} L_{vi} - (\delta + \mu) P_{vi}$$
$$\frac{dI_{vi}}{dt} = \phi\delta P_{vi} + \frac{\omega}{10} L_{vi} - (\tau + \mu) I_{vi}$$
$$\frac{dL_{vi}}{dt} = (1-\phi)\delta P_{vi} + \tau I_{vi} - \sigma\lambda_{vi} L_{vi} - \left(\frac{\omega}{10} + \mu\right) L_{vi},$$

where $\lambda_{vi} = (\kappa_i / \langle\kappa\rangle)\beta(\kappa_1 I_{v1} + \kappa_2 I_{v2})$. Figure 1C shows the time progression of disease incidence over the first 20 years of intervention, starting from two baseline incidences (1000 and 50 per 100,000 person-years). Figure S1 shows the impact of the same intervention for a range of baseline incidences, now at discrete time points (20 years after the beginning of the intervention and the asymptotic limit of infinite time). It is evident that intervention effectiveness is consistently lower when individual risk is heterogeneous (dash-dotted are consistently below dashes lines in Figures 1C and S1B).

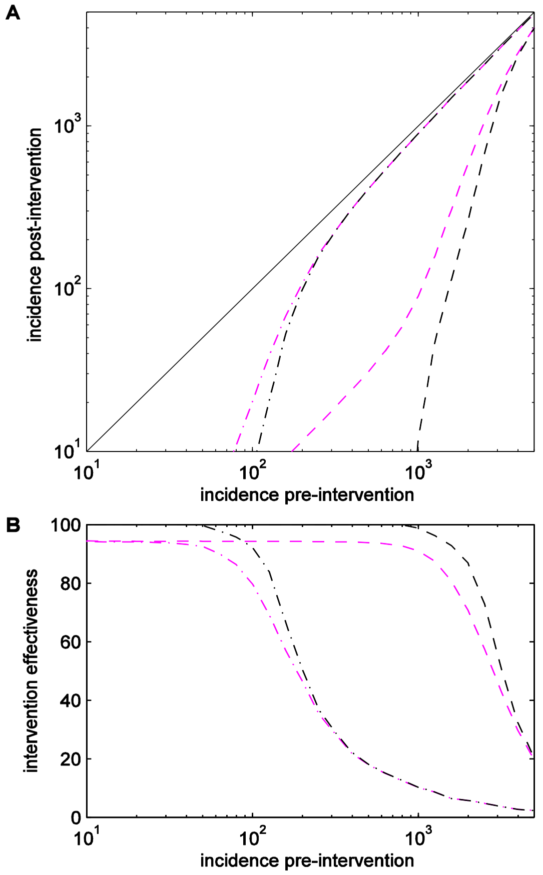

Figure S1. **Effectiveness at 20 years and equilibrium post-intervention.** This particular intervention reduces the force of infection upon naive individuals to $\sigma\lambda_i$ and the reactivation rate to $\omega/10$. **A**, Incidence post-intervention vs incidence pre-intervention. **B**, Intervention effectiveness calculated as $1 - I_v / I_c$, where $I_c = \sum_i I_i$ and $I_v = \sum_i I_{vi}$. Intervention analyses are represented by broken lines: magenta correspond to 20 years from the beginning of the intervention while black refer to equilibrium post-intervention; dash-dotted lines were generated by the heterogeneous model while dashed come from the mean field approximation (homogeneous).

References

1. Gomes MGM, Aguas R, Lopes JS, et al. How host selection governs tuberculosis reinfection. *Proc R Soc B* 2012; 279: 2473-8.
